# Physiological Responses of a Model Marine Diatom to Fast pH Changes: Special Implications of Coastal Water Acidification

**DOI:** 10.1371/journal.pone.0141163

**Published:** 2015-10-23

**Authors:** Yaping Wu, John Beardall, Kunshan Gao

**Affiliations:** 1 State Key Laboratory of Marine Environmental Science, Xiamen University, Xiamen, China; 2 School of Biological Sciences, Monash University, Clayton, Victoria, 3800, Australia; University of Connecticut, UNITED STATES

## Abstract

Diatoms and other phytoplankton in coastal waters experience rapid pH changes in milieu due to high biological activities and/or upwelled CO_2_-rich waters. While CO_2_ concentrating mechanisms (CCMs) are employed by all diatoms tested to counter low CO_2_ availability in seawater, little is known how this mechanism responds to fast pH changes. In the present study, the model diatom *Thalassiosira pseudonana* was acclimated for 20 generations to low pH (7.81) at an elevated CO_2_ of 1000 μatm (HC) or to high pH (8.18) at ambient CO_2_ levels of 390 μatm (LC), then its physiological characteristics were investigated as cells were shifted from HC to LC or vice versa. The maximal electron transport rate (ETR_max_) in the HC-acclimated cells was immediately reduced by decreased CO_2_ availability, showing much lower values compared to that of the LC-acclimated cells. However, the cells showed a high capacity to regain their photochemical performance regardless of the growth CO_2_ levels, with their ETR_max_ values recovering to initial levels in about 100 min. This result indicates that this diatom might modulate its CCMs quickly to maintain a steady state supply of CO_2_, which is required for sustaining photosynthesis. In addition, active uptake of CO_2_ could play a fundamental role during the induction of CCMs under CO_2_ limitation, since the cells maintained high ETR even when both intracellular and periplasmic carbonic anhydrases were inhibited. It is concluded that efficient regulation of the CCM is one of the key strategies for diatoms to survive in fast changing pH environment, e.g. for the tested species, which is a dominant species in coastal waters where highly fluctuating pH is observed.

## Introduction

In coastal waters, strong diel pH variations are often observed due to high biological production, upwellings or riverine input [1,2]. Superimposed on such natural pH variations, global oceans are being acidified due to continuously increasing atmospheric CO_2_ concentration and its subsequent increased dissolution, which will lead to an overall pH drop in the oceans of 0.3–0.4 units by 2100 [3,4]. In combination with eutrophication and hypoxia events, acidification may occur at a faster rate in coastal waters compared to open oceans [5]. Therefore, short-term pH fluctuations may become larger with progressive ocean acidification. Such changes in the chemical environment may affect cytosolic pH [6] (and references therein) and cell homeostasis [7–9]. Therefore, it is of significance to examine the physiological responses of marine organisms to rapid pH changes in addition to their adaptive and evolutionary responses.

Marine diatoms contribute about 40% of oceanic primary productivity, playing a key role in carbon export to deep ocean waters and consequent regulation of global climate change [10,11]. In addition, diatoms also play an important role in the marine food web due to their high abundance and wide size distribution [12]. Therefore, how diatoms respond to ocean acidification (OA) or multiple environmental forcings is of general significance. However, documented findings on the effects of OA have been controversial, showing OA either caused enhanced growth [13,14], had neutral effects or inhibited growth of diatoms [15]. As one of the most sensitive mechanisms to changes in pCO_2_, the CO_2_ concentrating mechanism (CCM) has been suggested to be associated with a range of physiological processes [16]; consequently down regulation of CCMs under OA could have beneficial or detrimental effects under different light levels or treatment regimes [17,18].

In coastal waters and upwelling areas with highly fluctuating pH, diatoms are often the dominant representative phytoplankton group [2]. In these areas, pH fluctuations can exceed the 0.4 units predicted for the end of the century (and the same is true for corresponding CO_2_ levels [2]. While they experience pH variations, the cells usually suffer from frequent CO_2_ limitation, over short time scales, that can be a selective pressure for phytoplankton with active CCMs [9]. It is still unclear how diatoms may regulate their physiology to respond to rapid changes in pH and related changes in carbonate chemistry, and maintain a balance between photosynthetic efficiency and energy cost [19]. CCM related genes have been shown to respond to CO_2_ changes within 1 hr when cyanobacteria were exposed to CO_2_-free conditions [20]. However, since the downstream syntheses of CCM components should take longer if changes in gene expression are involved, how fast physiological responses to changes in carbonate chemistry occur remains unknown.

Since diatoms might activate anti-stress mechanisms quickly to cope with the fast chemical changes in coastal waters, to maintain CO_2_ supply when cells encounter carbon shortage [21]. We therefore chose *Thalassiosira pseudonana*, a model diatom species frequently used for research in the past decades, to study whether diatoms can regulate physiological processes over a short timescale to respond to fast changes in pH/pCO_2_. This species is distributed in coastal waters as well as open oceans. When grown under OA conditions, the growth rate shows little response under low light [22,23] but suffers from inhibition under high light levels [17,24]. Here, we show that this diatom can modulate its photosynthetic physiology to quickly respond to pH/pCO_2_ changes, which may provide an advantage that allows it to outcompete other species in coastal waters.

## Materials and Methods

### Species and culture condition

*Thalassiosira pseudonana* (CCMP 1335), originally isolated from Moriches Bay, Long Island, USA, was inoculated in Aquil artificial seawater medium with a salinity of 35‰ [25], and grown in triplicate independent cultures (500 mL Erlenmeyer flasks) in a plant growth CO_2_ chamber (HP1000G-D, Ruihua) at 20 ± 0.1°C with a 14 h:10 h light: dark cycle. The cultures were illuminated at 120 μmol photons m^-2^ s^-1^ provided by cool white fluorescent lamps, and partially renewed with CO_2_ pre-equilibrated medium every day to maintain cell concentrations within a range of 8×10^4^ to 3×10^5^ cell mL^-1^. Target pH (*p*CO_2_) values in the cultures and the fresh medium were achieved by bubbling air (390 μatm) or pre-mixed air-CO_2_ mixtures (1000 μatm) within the plant growth chamber, which controlled the high CO_2_ level with a variation of less than 30 μatm. Cultures were acclimated under the respective CO_2_ level for nine days (~20 generations).

### Determination of seawater carbonate chemistry

pH was measured prior to and after the daily dilution as well as at the middle of the light period by a pH meter (pH510, Oaklon) which was calibrated with NBS buffer, to assure the stability of the carbonate system in cultures. Dissolved inorganic carbon (DIC) was sampled regularly and measured with an automatic system (AS-C3, Apollo SciTech Inc.) using an infrared gas analyzer (IRGA; Li-Cor7000, Li-Cor) after the samples were filtered into a syringe without any water/air CO_2_ exchange. Samples of culture medium (0.5 mL) were acidified using phosphoric acid, and any subsequently released CO_2_ was quantified by the IRGA. Parameters of the carbonate system were calculated as described in [13]. The carbonate chemical parameters were maintained at relatively stable levels, with pH changes between dilutions being less than 0.04 units (Table 1)

**Table 1:** The major parameters of seawater carbonate system under the present and projected CO_2_ levels. Data are the means ± SD of 6 measurements from triplicate bottles before and after dilution in a typical day.

parameter	LC-acclimated	HC-acclimated
pH	8.18±0.04	7.81±0.03
DIC (**μ**mol kg^-1^)	2023±28	2184±21
CO_2_ (**μ**mol kg^-1^)	12.6±1.4	33.6±2.4
HCO_3_^-^ (**μ**mol kg^-1^)	1828±19	2063±17
CO_3_^2-^ (**μ**mol kg^-1^)	182±17	87.7±9
pCO_2_ (**μ**atm)	390±41	1033±72

### Chlorophyll fluorescence measurements

To assess the photochemical responses of the cells to changes in the seawater carbonate chemistry, 50 mL sub-culture of LC- or HC-acclimated cultures was aseptically taken from each culturing bottle during the middle of the light period, and placed in a water bath to control temperature at ~20°C. 5 mL culture (LC-acclimated or HC-acclimated) was then dispensed into 20 mM Tris buffered medium with different pH levels (pH_NBS_, i.e. 7.82, 8.10, 8.37 and 9.50), and illuminated in the same growth chamber. Rapid light curves (RLCs) were then measured using a Xenon-Pulse Amplitude Modulated Fluorometer (XE-PAM, Walz). To assess the contribution of diffusive CO_2_ entry to photosynthetic efficiency, sub-samples were pipetted directly from illuminated cultures during the middle of light period, dispensed into 20 mM Tris buffered seawater medium that was pre-adjusted to target pH with or without ethoxyzolamide (EZ), a membrane permeable inhibitor of carbonic anhydrase, at a final concentration of 200 μmol L^-1^ (pre-tests showed that 150 μmol L^-1^ is a sufficient concentration).

The RLC was determined as relative electron transport rate (rETR) in response to eight different and increasing actinic PAR intensities, with a 10 s duration for each increment separated by a 0.8 s saturating pulse (4000 μmol m^-2^ s^-1^).

### Determination of activity of carbonic anhydrase

Cells grown under the two pCO_2_ levels were harvested by gentle filtration onto a polycarbonate membrane, washed and re-suspended (cell concentration around 4×10^6^ mL^-1^) in DIC free seawater that was buffered with 20 mM barbitone at pH 8.4. The CA_e_ (external CA) and CA_total_ (including periplasmic and intracellular CA) activity of the intact cells was determined by an electrometric method according to [26]. A 5 mL cell suspension (for CA_ext_) or cell suspension disrupted with a sonicator (102C Branson, for CA_total_) with amplitude set at 30%, then added to a reaction cuvette and stirred. The disruption of cells was confirmed under a microscope. The time required for the pH to drop from 8.2 to 7.2 after the addition of 2 mL ice-cold CO_2_-saturated pure water was recorded. The temperature during the reaction was controlled at 4°C.

### Calculations and statistical analysis

The rETR was calculated as: rETR = Ф_PSII_×0.5×PFD (μmol e^-^ m^-2^ s^-1^), where Ф_PSII_ is the photochemical quantum yield of PSII in light, PFD is the actinic PAR intensity (μmol photons m^-2^ s^-1^), and the factor 0.5 accounts for approximately 50% of all the absorbed energy being allocated to PSII. Rapid light curves were fitted according to [27] as follows:

y = x / (ax^2^ + bx + c), Where y is the rETR, x is the photon flux density of actinic light (μmol photons m^-2^ s^-1^), a, b and c are the adjustment parameters. P_m_, α and E_k_ was calculated as: ${\text{P}}_{\text{m}}\;=\;1/(\text{b}+2\sqrt{\text{ac}})$, α = 1/c, E_k_ = P_m_/a

Activity of the carbonic anhydrase (in enzyme units, EU) was calculated as follows:

10×[(T_b_/T_c_)-1] / 10^6^ cells, where T_b_ and T_c_ were the times in seconds for the pH drop with or without cells, respectively.

Statistical differences among treatments were tested with one-way ANOVA using a Tukey test by Origin 8.0, and the significance level was set at p = 0.05.

## Results

**Fig 1:**
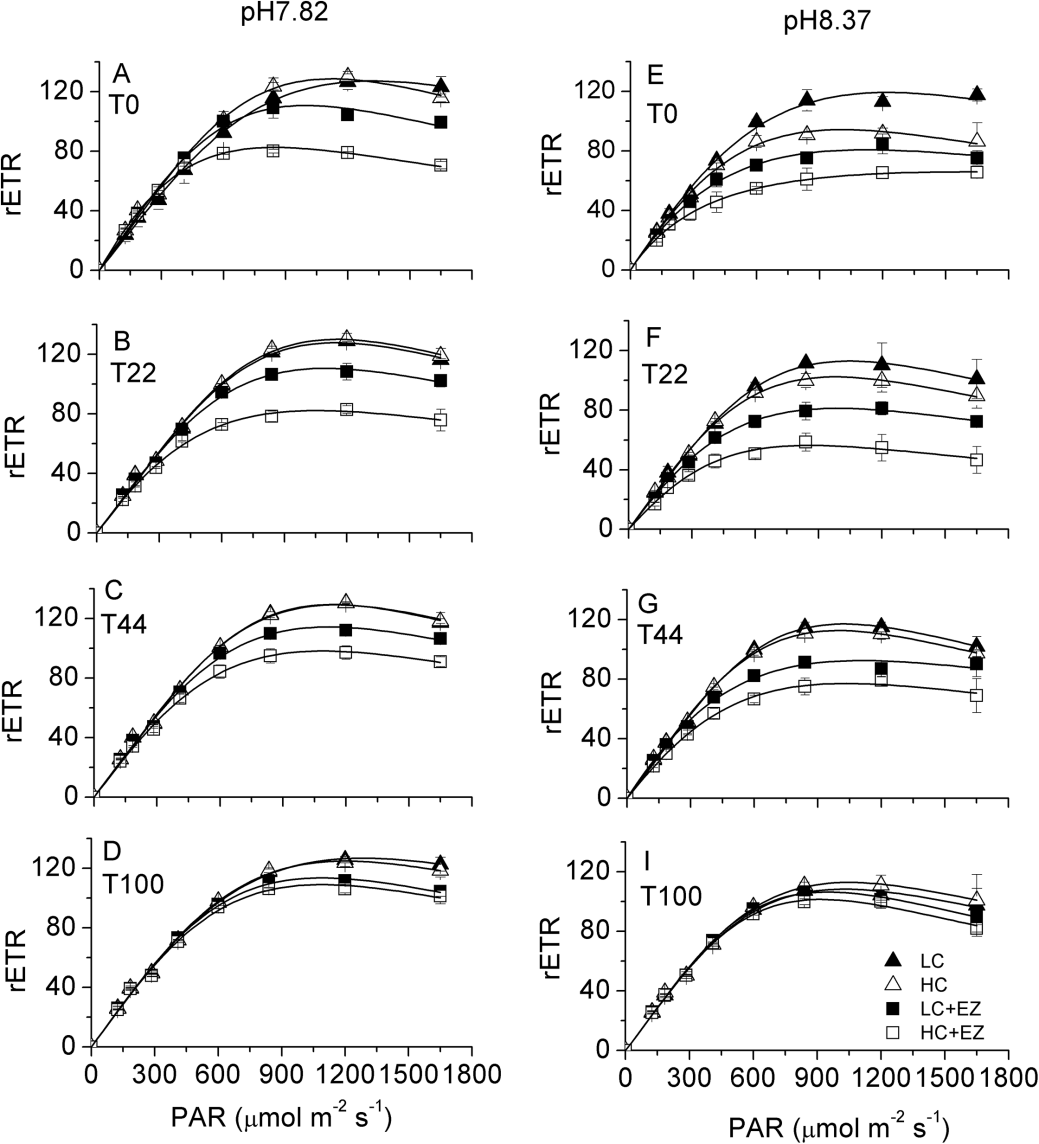
The rapid light curves of LC-acclimated and HC-acclimated grown cells in the presence or absence of EZ measured in pH 7.82 (A, B, C, D) or pH 8.37 (E, F, G, I) buffered medium(Tris,20 mM) at different times (T_0_,T_22_,T_44_ and T_100min_). Vertical bars represent SD, n = 3.

The rapid light curves (RLCs) showed typical patterns of photosynthesis versus irradiance; relative electron transport rate (rETR) increased with increasing light (1), while significant inhibition of rETR by EZ was observed for RLCs measured at both pH 7.82 and pH 8.37 (Fig 1). rETR values of LC-acclimated and HC-acclimated cells treated with EZ (LC-acclimated +EZ or HC-acclimated +EZ cells) were significantly lower than those of the controls (without EZ) (p<0.001), and recovered partially after 22–100 min incubation. The rETR of LC-acclimated cells was similar to that of HC-acclimated cells when measured at pH 7.82 (Fig 1A–1D), while when samples were assayed at pH 8.37, the rETR of HC-acclimated cells were lower than that of LC-acclimated cells in the first 22 min (Fig 1E and 1F), but achieved similar values after 44 min incubation (Fig 1G and 1I). After 100 min incubation, rETR values of EZ treated samples was still lower than the control treatments when assayed at pH 7.82, while no significant differences were found among samples that were assayed at pH 8.37. Similar responses were observed at pH 8.10; rETR rates were significantly inhibited by EZ but recovered partially during the incubation (Fig A–D in S1 Fig) (p<0.001). However the responses were completely different at pH 9.50 where essentially all the DIC was available only as HCO_3_^-^. Here no differences were found among treatments, and no further recovery occurred during the incubation (Fig E–I in S1 Fig).

**Fig 2:**
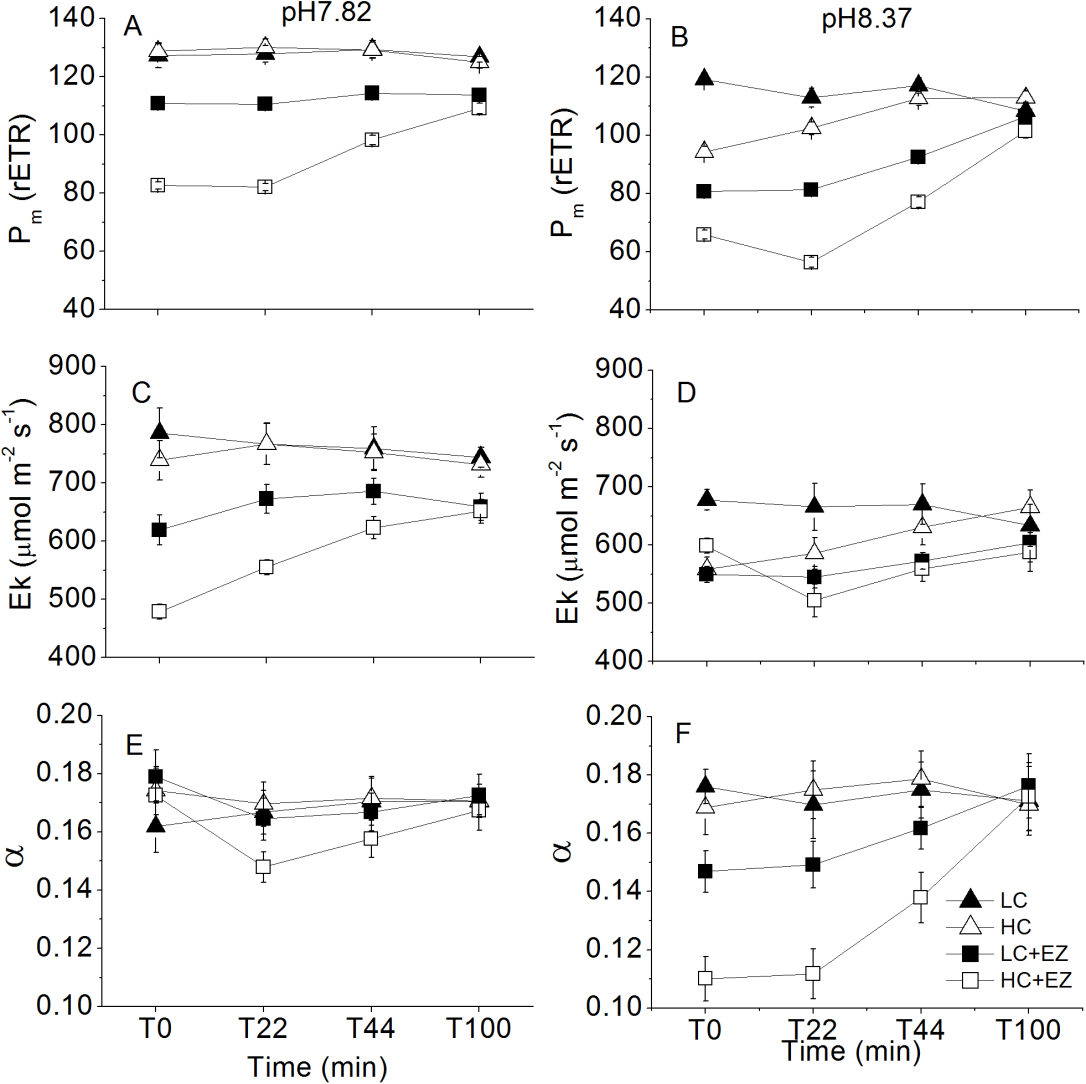
The time course of photosynthetic parameters (P_m_, the maximal rETR; E_k_, the light saturation point and α, the light utilization efficiency) of LC-acclimated and HC-acclimated rapid light curves in the presence or absence of EZ at pH 7.82 (A, C, E) or pH8.37 (B, D, F), Vertical bars represent SD, n = 3.

The maximal relative electron transport rate (rETR_m_) measured at pH 7.82 remained relatively stable for LC-acclimated and HC-acclimated cells, values for both being around 128±4 μmol e^-^ m^-2^ s^-1^ during the 100 min incubation (Fig 2A). The rETR_m_ of LC-acclimated +EZ cells showed a similar pattern, with relatively lower values around 113±2 μmol e^-^ m^-2^ s^-1^. While the rETR_m_ of HC-acclimated +EZ cells was around 73% of that of LC-acclimated +EZ cells for the first 22 min, then recovered quickly to similar values as LC-acclimated + EZ cells for the subsequent 78 min of incubation (Fig 2A). There were obvious differences between LC-acclimated and HC-acclimated cells at pH 8.37 when CO_2_ availability was only 25% of that at pH 7.82 (Fig 2B). rETR_m_ values of LC-acclimated cells measured at pH 8.37 decreased slightly but significantly during the 100 min incubation (p<0.01), while HC-acclimated cells had rETR_m_ values significantly lower (by 20%) than those of LC-acclimated cells at T_0_ (p<1E-4), but increased linearly to reach similar values to those of LC-acclimated cells by the end of the incubation. The rETR_m_ values of HC-acclimated +EZ cells were 19% lower than those of LC-acclimated +EZ cells, but during the incubation, all increased gradually to final values that were close to those of non EZ treated samples (Fig 2B).

The E_k_ values of LC-acclimated and HC-acclimated cells at pH 7.82 remained relatively stable during the incubation, at 780±43 μmol m^-2^ s^-1^. The E_k_ of LC-acclimated +EZ cells was 619±26 μmol m^-2^ s^-1^ initially but increased slightly to 672±24 μmol m^-2^ s^-1^ then remained stable for the subsequent incubation (Fig 2C) (p = 0.058). The E_k_ of HC-acclimated +EZ cells was the lowest at T_0_, being around 77% of that of LC-acclimated +EZ cells, but increased gradually and reached a similar value to that found in LC-acclimated +EZ cells at the end of incubation (Fig 2C). The E_k_ measured at pH 8.37 remained relatively stable for the whole incubation at around 500–650 μmol m^-2^ s^-1^ with some variations but no clear trend (Fig 2D). The values of light utilization efficiency (α, initial slope of RLC) of all treatments were around 0.18 e^-^ photon^-1^ at T_0_ under pH 7.82, and remained stable in most treatments for the subsequent incubation (Fig 2E). While values for HC-acclimated +EZ cells decreased significantly from 0.17±0.009 to 0.15±0.005 e^-^ photon^-1^ at T22 (p<0.05), and then increased gradually to a similar value as the other treatments (Fig 2E). α of LC-acclimated and HC-acclimated cells at pH 8.37 was similar as to that at pH7.82, and remained stable for the whole incubation period, at around 0.17, though α of LC-acclimated +EZ and HC-acclimated +EZ cells was lower at T_0_, at 0.15±0.007 and 0.11±0.008 e^-^ photon^-1^, respectively (Fig 2F). While EZ treated cells all gradually increased to a similar value to that found in non-EZ samples at T_100_. RLC parameters measured at pH 8.10 and pH 9.50 are shown in S2 Fig.

**Fig 3:**
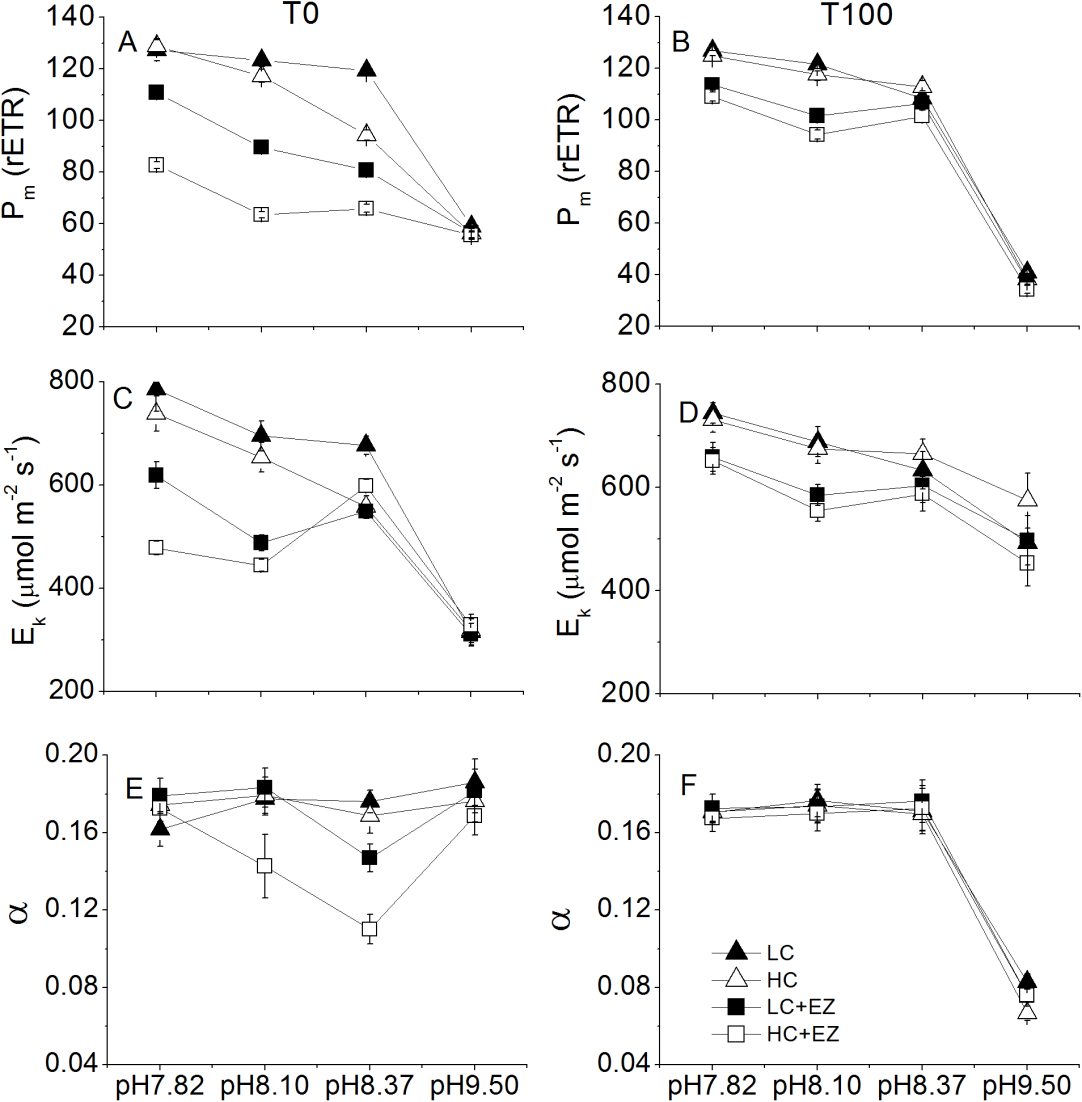
The maximal rETR, saturation point, light utilization efficiency of LC-acclimated and HC-acclimated grown cells measured immediately (A, C, E) or after 100 min incubation (B, D, F) in the presence or absence of EZ at various pH values (7.82, 8.10, 8.37 and 9.50). Vertical bars represent SD, n = 3.

The rETR_m_ values across the assay pH showed a decreasing pattern with increasing pH levels, at T_0_ (Fig 3A), rETR_m_ values of LC-acclimated cells was similar to those of HC-acclimated cells at pH 7.82. While HC-acclimated values were lower than those of LC-acclimated at higher pH, around 80% of LC-acclimated at pH8.37, but all decreased to the same value, around 60 μmol e^-^ m^-2^ s^-1^, when measured at pH 9.50. rETR_m_ of HC-acclimated EZ cells was lower than LC-acclimated EZ, except for at pH 9.50 where both decreased to similar values to those of LC-acclimated or HC-acclimated cells. At T_100_, rETR_m_ of LC-acclimated and HC-acclimated cells were similar to those at T_0_, and dropped from 125±2 to 38±2 μmol e^-^ m^-2^ s^-1^ when the assay pH gradually increased from 7.82 to 9.50. rETR_m_ values of EZ treated cells were slightly lower than for non EZ samples, and decreased with increasing pH to a value of 38±2 μmol e^-^ m^-2^ s^-1^ at pH 9.50. The light utilization efficiency values of LC-acclimated and HC-acclimated cells were similar and stable, around 0.18 e^-^ photon^-1^, while the EZ treated samples had lower values at pH8.10 and pH8.37, but were similar to non-EZ samples at pH7.82 or pH9.50 (Fig 3E and 3F). The light utilization efficiency values at T_100_ were similar and stable for all treatments at pH 7.82 to pH 8.37, while they decreased sharply by 50% at pH 9.50. No differences were found among CO_2_ or EZ treatments at T_100_.

**Fig 4:**
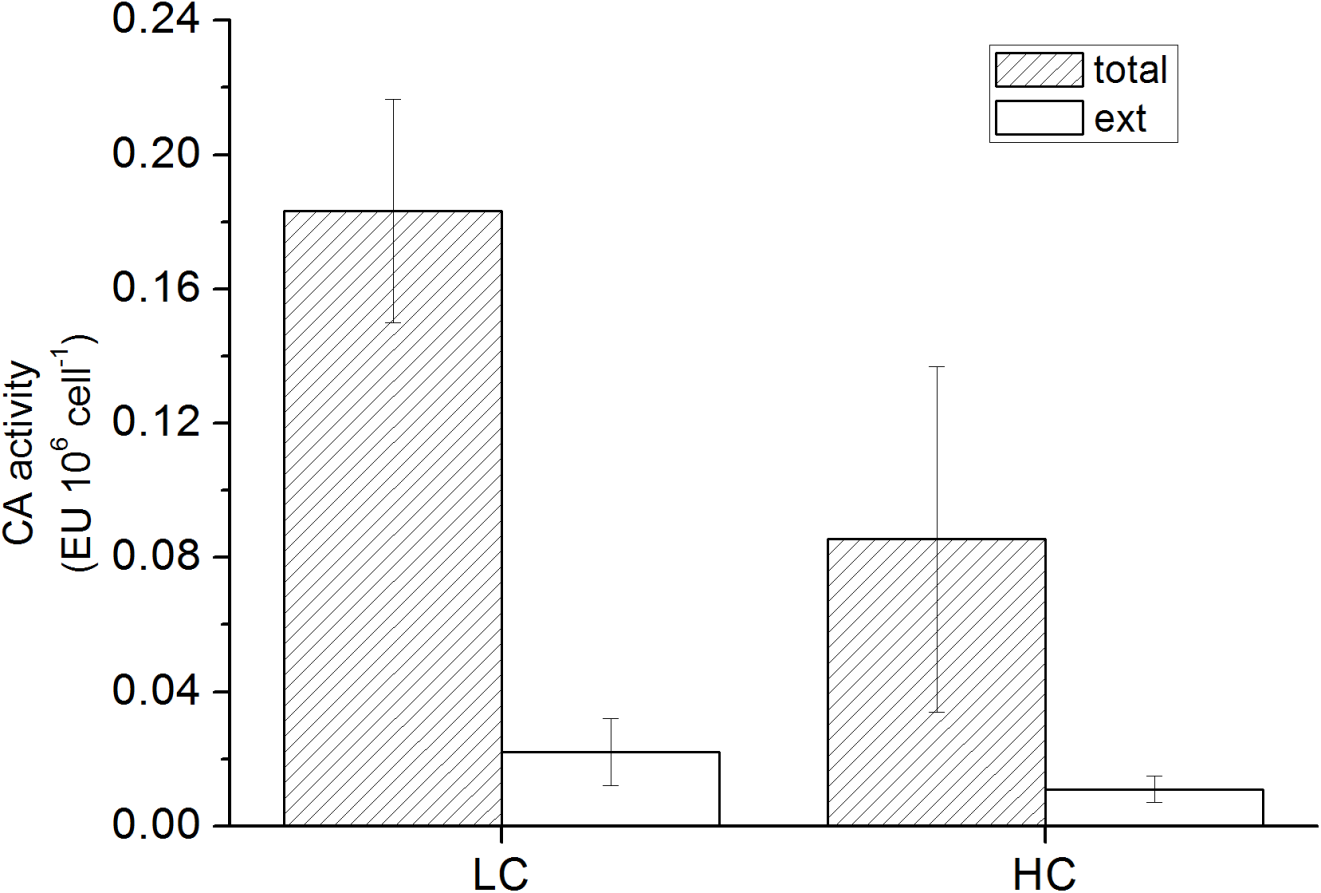
The external and total carbonic anhydrase activity of LC-acclimated and HC-acclimated grown cells. Vertical bars represent SD, n = 3.

The whole cell carbonic anhydrase activity (CA_total_) was 0.18±0.03 EU (10^6^ cells)^-1^ for LC-acclimated cells, while that of HC-acclimated cells was much less (p<0.01), around 0.09±0.05 EU (10^6^ cells)^-1^ (Fig 4B). Extracellular carbonic anhydrase (CA_e_) activity was detectable but very low for both LC-acclimated and HC-acclimated cells, at 0.02(±0.01) and 0.01 (±0.004) EU (10^6^ cells)^-1^ respectively.

## Discussion

Fluctuations of environmental factors are regarded as providing selection pressure for dominant species of phytoplankton [28]. As the oceans continue to absorb anthropogenic CO_2_, the buffering capacity of seawater will decrease, therefore phytoplankton cells are likely to experience increasing acidic stress with progressive ocean acidification [5,29]. This will be superimposed on the rapid and significant fluctuations in pH and CO_2_ concentration experienced by phytoplankton in upwelling regions and near-shore or estuarine environments[2]. In the present study, by comparing the physiological responses of a diatom, grown at two *p*CO_2_ levels, to rapid changes of pH, we concluded that *T*. *pseudonana* could respond quickly to changes of pH, to maintain a steady state supply of CO_2_. When LC-acclimated cells are exposed to high pH (limited CO_2_) environments, they could immediately achieve a relatively high electron transport rate, while there was an obvious lag before HC-acclimated cells responded to high pH. This may provide this diatom with a competitive advantage in coastal waters [30].

Short-term exposure to low pH/high CO_2_ should lead to negligible enhancement of photosynthesis of the LC-acclimated cells due to a sufficient supply of CO_2_ by the CCM [31], recognizing that in *T*. *pseudonana* a biochemical CCM based on C4 photosynthesis [32–36], rather than the biophysical CCM found in most other microalgae [31] may operate. The diatom tested in this study, when acclimated to LC conditions, can achieve higher electron flow than HC-acclimated cells [13], which could be attributed to the higher uptake of CO_2_ for the photosynthetic machinery [37]. Once the whole cellular enzymatic conversion between CO_2_ and HCO_3_^-^ was blocked by the inhibitor EZ, the rETR sharply decreased due to an inhibited CO_2_ supply; in such a case, the photosynthetic rate should solely depend on diffusive entry or active uptake of CO_2_. The higher rETR of LC-acclimated +EZ cells compared to that of HC-acclimated +EZ cells, suggests that when CO_2_ was the only source of inorganic carbon, LC-acclimated cells had a higher efficiency in active CO_2_ uptake. In *T*. *pseudonana*, the C4 pathway could play an important role during the acclimation under low CO_2_ or the diel cycle [32], since previous studies have demonstrated that *T*. *pseudonana* has the ability to concentrate CO_2_ via the C4 (or a C3-C4 intermediate) pathway for photosynthesis [33,34]. Indeed, our results are in agreement with a recent study, which revealed that C4 pathway-related genes were up-regulated during an acclimation under reduced CO_2_ availability [35], indicating a fundamental role for the C4 pathway in *T*. *pseudonana* photosynthesis, especially in a low CO_2_ environment [36].

The diminishing differences of rETR_m_ with time among the treatments with or without EZ in HC-acclimated or LC-acclimated cells (Fig 2A and 2B), indicated that there was an inducible mechanism operational for the carbon uptake once the cells encountered CO_2_ shortage [34,36], especially for EZ treated cells, and that this allowed recovery of photosystem II. Since light energy is essential for the operation of the CCM [38,39], when exposed to high pH with limited supply of CO_2_, the cells have to allocate extra energy for carbon transport or synthesis of C4 compounds, resulting in a lower light utilization efficiency (α) (Fig 2F). Even when major components of CCMs, extra- and intracellular carbonic anhydrase, were blocked with sufficient inhibitor, rETR_m_ values of EZ cells could recover by ~40% to ~90% of non-EZ treated samples within 100 min, indicating that the cells could modulate their physiological processes to adapt to extreme conditions[36], including e.g. the up-regulation of the C4 pathway, reallocation of light energy to CCM, active CO_2_ uptake, to maintain steady state CO_2_ supply [19].

Phytoplankton experience fluctuating environmental changes due to their temporal-spatial distributions. Riverine input, mixing or cyclones could stimulate the growth of some species which have strong nutrient accumulating mechanisms [40,41], while fluctuating sunlight would influence photosynthetic carbon fixation in surface seawater depending on levels of solar radiation [42,43] and phytoplankton cells circulating in the water column will be exposed to rapidly changing light levels. Therefore, phytoplankton species may have acquired, during evolution, sophisticated mechanisms to modulate their physiology to respond to fast environmental changes. As revealed recently, the chain forming diatom could maintain photochemical performance, even exposed to acute pH changes [44]. CO_2_ is the major factor regulating CCM activity; while some diatoms rely on CO_2_ diffusion more than active transport of bicarbonate across the plasma membrane [45], the varied CO_2_ availability in coastal waters may affect their photosynthesis. Based on the findings of this work, we see that diatoms like *T*. *pseudonana* can cope with frequent fluctuations of pH/CO_2_, and maintain CO_2_ supplies at a steady state for photosynthesis. This can be considered as an advantage for these species, allowing them to dominate coastal waters [46].

## Supporting Information

S1 Fig The rapid light curves of LC-acclimated and HC-acclimated grown cells in the presence or absence of EZ that were measured in pH 8.10 (Fig A, B, C, D) or in pH 9.50 (Fig E, F, G, I) buffered medium(Tris,20 mM)and at different time (T_0_,T_22_,T_44_ and T_100min_). Vertical bars represent SD, n = 3.
(TIF)

S2 Fig The time course of photosynthetic parameters (P_m_, the maximal rETR; E_k_, the light saturation point and α, the light utilization efficiency) of LC-acclimated and HC-acclimated rapid light curves in the presence or absence of EZ at pH 8.10 (Fig A, C, E) or pH9.50 (Fig B, D, F), Vertical bars represent SD, n = 3. (TIF)
