# Supplementary figures and images for: Physiological Responses of a Model Marine Diatom to Fast pH Changes: Special Implications of Coastal Water Acidification
Source: PLoS One. 2015 Oct 23;10(10):e0141163. doi: 10.1371/journal.pone.0141163 (PMC4619668; doi:10.1371/journal.pone.0141163)

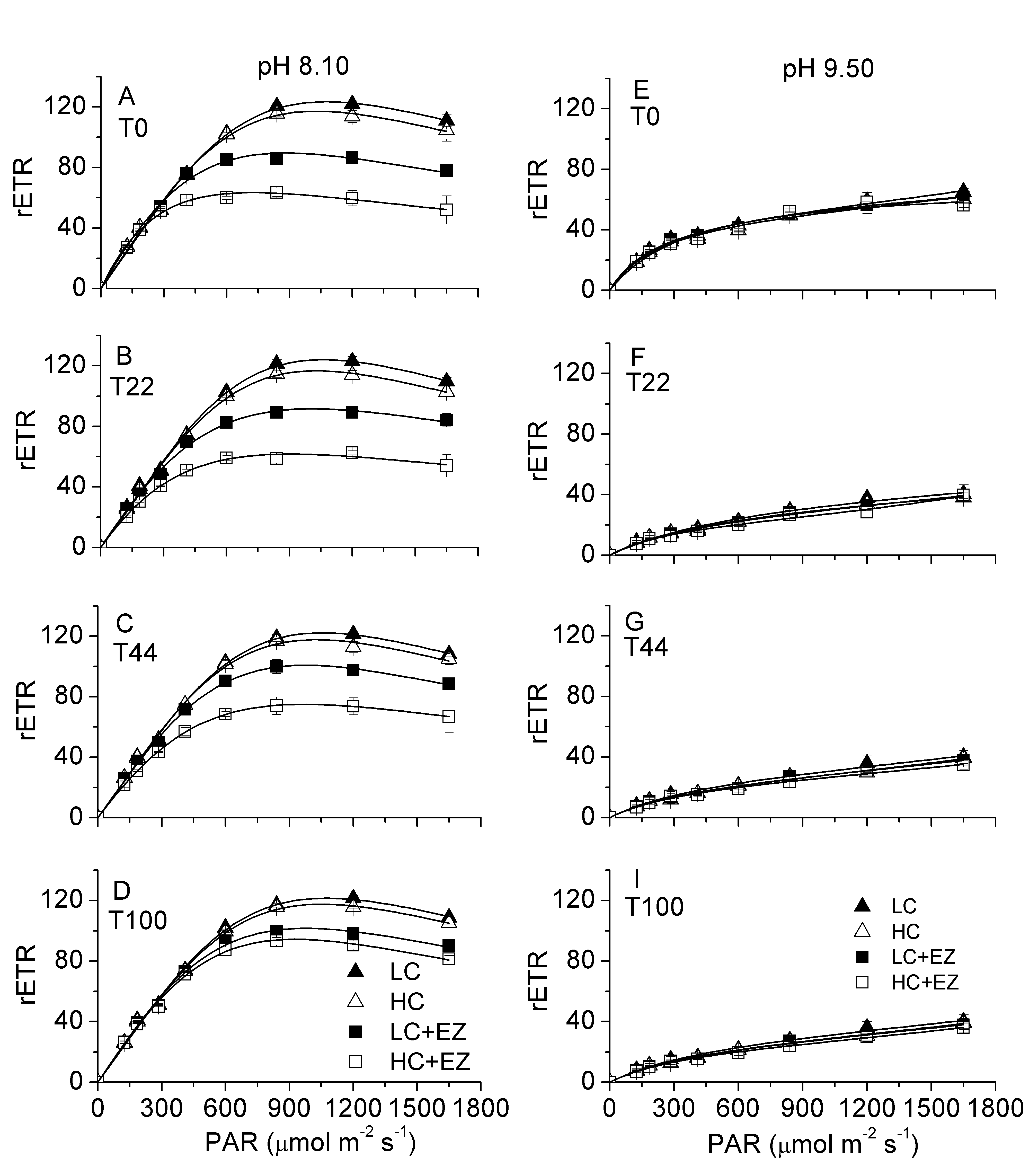

Supplement: S1 Fig — Vertical bars represent SD, n = 3. (TIF) [file pone.0141163.s001.tif]

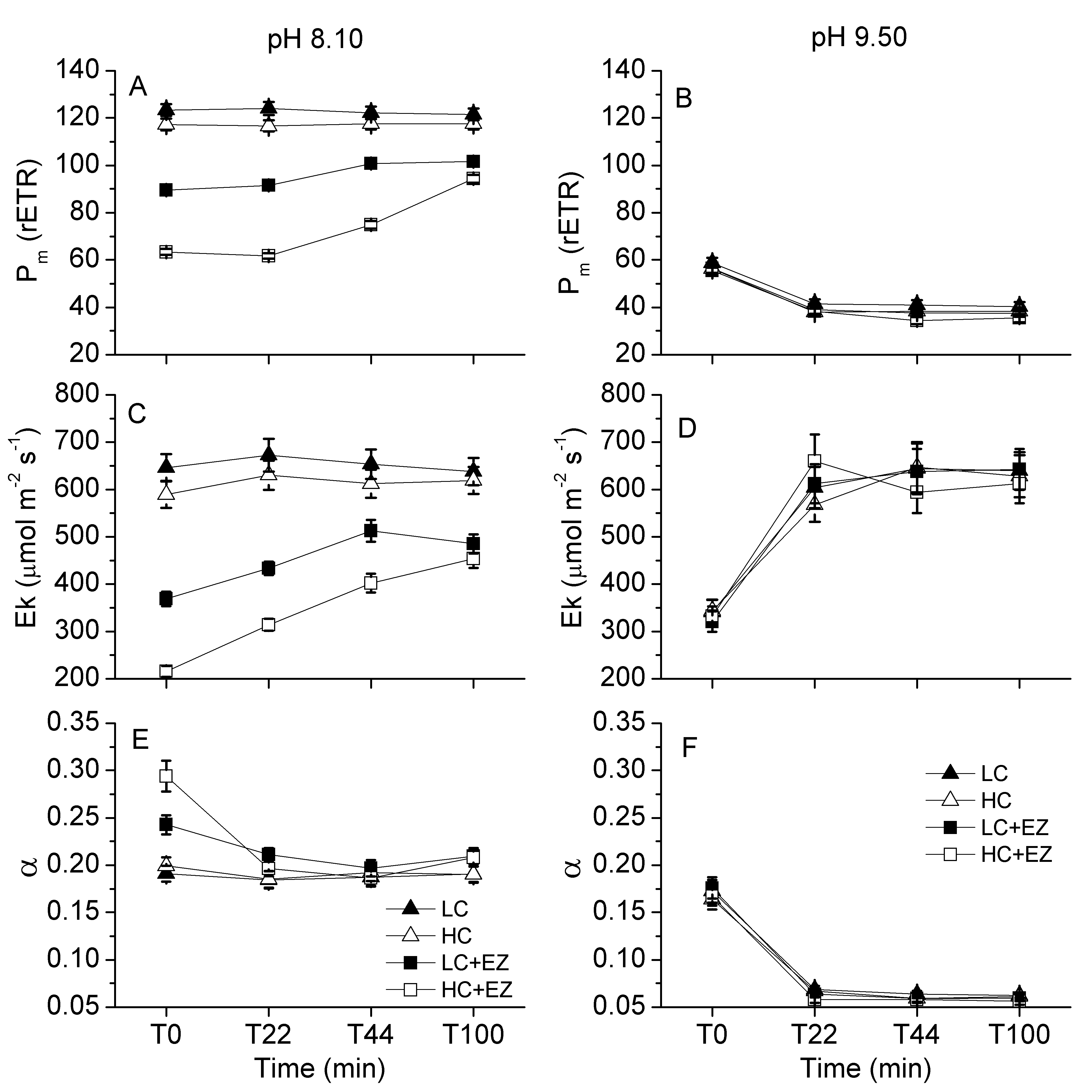

Supplement: S2 Fig — (TIF) [file pone.0141163.s002.tif]
